# Developing training programmes for eye teams

**Published:** 2018-07-31

**Authors:** Lynn Anderson, Karl Golnik

**Affiliations:** 1 CEO: International Joint Commission on Allied Health Personnel in Ophthalmology (IJCAHPO), Minnesota, USA.; 2 Chairman: Department of Ophthalmology, University of Cincinnati, ICO Director for Education, IJCAHPO Secretary for International Affairs, Cincinnati, Ohio, USA.

With the increased need for eye care services worldwide, educators must approach curriculum design and teaching in a systematic way with a clear goal in mind: education of the whole eye care team, with specific competencies, to work together effectively to provide high quality patient care.

**Figure 1:**
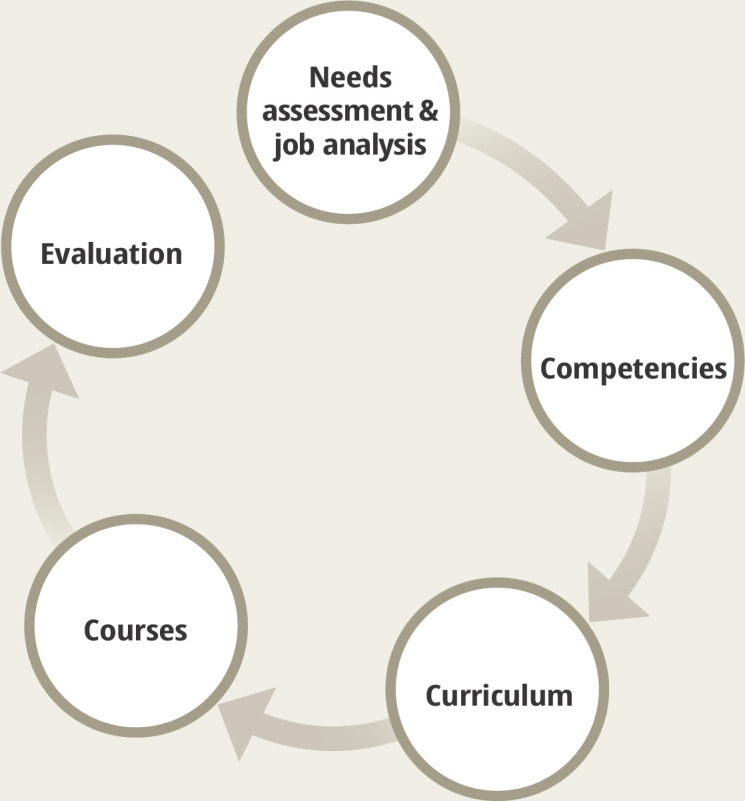
Developing appropriate and relevant training programmes.

Developing or adapting training programmes for the different personnel in the eye team follows the cycle shown in Figure 1.

The first task is to perform a **needs assessment** (i.e., which key competencies must the eye team be able to perform in order to ensure high quality care for patients?) and a **job analysis** (i.e., which roles are best suited to take responsibility for the required tasks?). Shifting tasks appropriately can improve productivity.

**Competencies.** The various tasks required by each team member can be broken down into specific ‘competencies’ (i.e. a specific task, performed in a defined way and to a specific standard). Developing training for each competency is based on:

1. Knowledge: What must they know?
2. Skills: What must they be able to do?
3. Attitudes: What motivates them to learn and perform?

The answers form the ‘intended learning outcomes’ determines *what* and *how* they should be taught and assessed (the **curriculum**) and then *organised* within manageable building blocks (as **courses** or **modules**).

The final component is **evaluation** of the training programme to check whether it had enabled eye care personnel to attain the relevant competencies. Internationally established standards of practice and assessment, such as IJCAHPO's core progressive and specialty certifications and ICO's core curriculums, facilitate a standardised approach to guide training programmes.

As the need for eye care changes and develops over time in a particular population, the cycle is used to revise and review the curriculum and competencies.

